# Revealing the Central Mechanism of Acupuncture for Primary Dysmenorrhea Based on Neuroimaging: A Narrative Review

**DOI:** 10.1155/2023/8307249

**Published:** 2023-02-18

**Authors:** Benlu Chen, Qin Guo, Qiwen Zhang, Zhong Di, Quanai Zhang

**Affiliations:** ^1^The Third School of Clinical Medicine, Zhejiang Chinese Medical University, Hangzhou, China; ^2^Department of Acupuncture and Moxibustion, The Third Affiliated Hospital of Zhejiang Chinese Medical University, Hangzhou, China

## Abstract

**Objective:**

The central mechanism of acupuncture for primary dysmenorrhea was explored by summarizing the changes in different regional networks of the brain induced by acupuncture stimulation by analyzing the existing studies.

**Methods:**

The original studies were collected and selected from three English databases such as PubMed and four Chinese databases as China Knowledge Network (CNKI). The main keyword clusters are neuroimaging, acupuncture, and primary dysmenorrhea.

**Results:**

The literature review yielded 130 possibly qualified studies, and 23 articles fulfilled the criteria for inclusion. Regarding the type of acupuncture studies, 6 moxibustion studies and 17 manual acupuncture studies for primary dysmenorrhea were included. Based on functional magnetic resonance imaging (fMRI), perfusion-weighted imaging (PWI), and positron emission tomography-computer tomography techniques (PET-CT), one or more analysis methods such as amplitude of low-frequency fluctuations (ALFF), regional homogeneity (ReHo), functional connectivity (FC), and independent components analysis (ICA) were used. The results are summarized. To summarize the high-frequency brain area alterations observed in patients with acupuncture-induced primary dysmenorrhea were the anterior cingulate gyrus, thalamus, insula, precentral gyrus, middle frontal gyrus, postcentral gyrus, putamen, and cerebellum.

**Conclusion:**

The results suggest that the mechanism of acupuncture in the treatment of primary dysmenorrhea is the involvement of networks regulating different areas of the brain in the analgesic effects of acupuncture. The brain regions involved in primary dysmenorrhea acupuncture analgesia were mainly located in the pain matrix, default mode network, salience network, and limbic system.

## 1. Introduction

Primary dysmenorrhea (PD) is defined as dysmenorrhea without organic disease of the reproductive organs and accounts for more than 90% of the incidence of dysmenorrhea [1]. Approximately 45% to 90% of women experience dysmenorrhea [2]. Between 10% to 25% suffer from severe dysmenorrhea, which seriously affects the quality of life of patients [3]. Existing studies suggest that the pathogenesis of dysmenorrhea is due to elevated secretion of Prostaglandin F2a (PGF2*α*) and Prostaglandin E2 (PGE2) causing contraction of uterine vessels and muscles, increased uterine activity, excessive contracture resulting in reduced blood flow and tissue ischemia, and hypoxia causing pain [2, 4]. Current treatments for dysmenorrhea include pharmacotherapy and complementary drug therapy. The common pharmacological therapies are oral nonsteroidal anti-inflammatory drugs (NSAIDs) and birth control pills. However, long-term use of drugs can produce many gastrointestinal side effects. In supplemental medicine, herbs, yoga, relaxation, psychotherapy, massage, and acupuncture are used for healing [5].

Acupuncture is commonly used as an alternative treatment for PD because of its efficacy and safety [6]. Available studies have indicated that acupuncture treats PD by regulating endocrine and analgesic substances, modulating immune-inflammatory responses, improving uterine blood flow, and reducing oxidative stress [7].

There are more neuroimaging research methods, which are commonly used to study functional brain connectivity, metabolite content, neuroreceptor distribution, and brain area activation, mainly fMRI and PET. fMRI is a blood oxygenation level-dependent functional brain imaging, which is based on the principle of measuring changes in the degree of oxygenation in local cerebral blood flow, which indirectly reflects the operational activities of brain regions [8]. There are several analysis methods for fMRI, and the commonly used analysis methods are ALFF/FC/ReHo. ALFF measures the amplitude of fluctuations at low frequencies when the nerve activity is stationary [9, 10]. FC reveals the exchange of functional information between different brain regions at the anatomical level [11, 12]. ReHo detects localized regional functional synchronization in the brain, responding to the state of neural activity [13]. PET imaging is a nuclear medicine imaging technique in which a radioactive tracer is usually injected intravenously, followed by observation of the distribution and quantification of the target substance. PET imaging is commonly used to detect cerebral blood flow, assess functional indicators such as glucose metabolism and oxygen consumption, and quantify differences in the density of proteins such as receptors, transport proteins, and enzymes.

Neuroimaging has been widely used to observe brain alterations in patients with dysmenorrhea, and studies have shown that patients with PD have a particular alteration in the density of functional connectivity in brain networks associated with pain [14, 15]. Some studies have also attempted to clarify the targets of the action of acupuncture for PD through neuroimaging [16]. Or use neuroimaging for the prediction of the onset of dysmenorrhea [17–19]. There is a lack of reviews to summarize the central mechanisms of acupuncture for PD and to assess the brain alterations in people with PD under acupuncture intervention. This review aims to further clarify the central mechanism of acupuncture for PD by systematically reviewing 25 studies on neuroimaging of acupuncture for PD, analyzing the findings and methodological issues and changes in brain networks, and providing a reference for future research directions [5].

## 2. Methods

### 2.1. Inclusion Criteria

The inclusion criteria were as follows:

- Study types: published randomized and nonrandomized controlled studies of acupuncture for PD in English and Chinese
- Participants: individuals were diagnosed with PD
- Intervention: acupuncture or moxibustion, and neuroimaging techniques were used to study acupuncture-induced alterations in the brain

### 2.2. Exclusion Criteria

The exclusion criteria were as follows:

- Duplicate article or incomplete article
- Review, case report, and protocol
- Animal research

Flowchart for the literature selection and search process (Supplementary Figure 1).

### 2.3. Search Strategy

Data were collected from three English databases (PubMed, Embase, and Cochrane Library) and four Chinese databases (China Knowledge Network; CBM, Chinese Database; Chongqing VIP Database; and Wanfang Database) from the start of the database to 16 February 2022. The search terms are as follows: neuroimaging, acupuncture, and primary dysmenorrhea. A retrospective search of references included in the article was conducted. The search formula is shown in (Supplementary Table 1).

### 2.4. Data Extraction and Analysis

We extracted the following information: year of publication, and corresponding author's institution (Supplementary Table 2). Study design (timing of image acquisition, intervention modality, type of control, and clinical scale for PD) (Supplementary Table 3). Relevant neuroimaging information (scanning techniques, methods of analysis of data, and brain area outcomes of PD treated with acupuncture analgesia) (Supplementary Table 4). Study details (manipulation, sample size, acupoint, treatment, and scanning results) (Supplementary Table 5). The previous was screened by two reviewers and differences were settled by consulting the third reviewer.

The Cochrane Risk of Bias tool (ROB2, ROBINS-I) was used to evaluate bias [20, 21] (Supplementary Figure 2).

## 3. Results

This review includes 23 original articles from a total of 25 studies.

### 3.1. Basic Information about the Study

**Figure 1:**
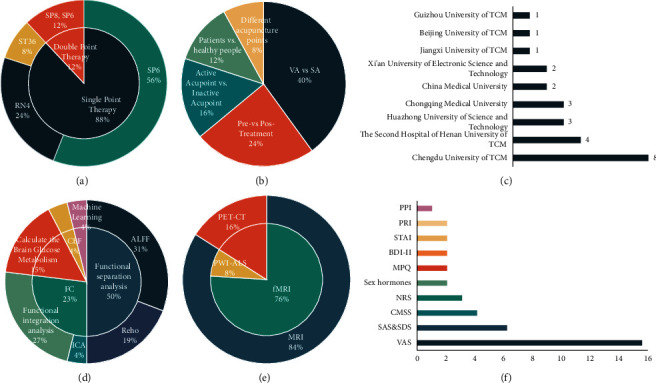
The study design included (a) the proportion of different types of acupuncture points, (b) the proportion of the type of control of PD, (c) the proportion of institutions, (d) the proportion of analysis methods of neuroimaging data, (e) the proportion of scanning techniques, and (f) the proportion of clinical scale of PD. VA: verum acupuncture; SA: sham acupuncture; CBF: cerebral blood flow; ALFF: amplitude of low-frequency fluctuations; FC: functional connectivity; ReHo: regional homogeneity; ICA: independent components analysis; PET-CT: positron emission tomography-computed tomography; MRI: magnetic resonance imaging; fMRI: functional magnetic resonance imaging; PWI: perfusion-weighted imaging; ASL: arterial spin labeling; PPI: present pain intensity; PRI: pain rating index; STAI: state-trait anxiety inventory; BDI-II: beck depression inventory-II; MPQ: McGill pain questionnaire; NRS: numerical rating scale; CMSS: cox menstrual symptom scale; SAS: self-rating anxiety scale; SDS: self-rating depression scale; VAS: visual analog scale.

The top one was Chengdu University of Traditional Chinese Medicine (8 studies) [17, 22–28] (Figure 1(c)).

11 RCT studies were included in the study, 4 were considered “low risk” [24, 28–30], and the remaining 7 were considered “some concerns” due to “deviations from the intended intervention” and “missing outcomes” [17, 22, 25, 26, 31–33]. Of the 14 non-RCT studies included, 3 were considered “low risk” [23, 27, 34], and the remaining 11 were considered “some concerns” due to “selection bias in participant studies,” “deviations from intended interventions,” and “missing outcomes” [35–42].

In this review, 6 studies (24%) were set up as a self-control model (pre- vs. post-treatment) [23, 35, 37, 38, 41, 43], 10 studies (40%) explored the response between real acupuncture (VA) and sham acupuncture (SA) in the brain [17, 28–30, 32–34, 39, 42], 4 studies (16%) discussed the difference between the neuroimaging of stimulated acupoints and the nonmeridian nonacupuncture group [22, 24–26], 3 studies (12%) explored the differences in neuroimaging between healthy individuals and patients [27, 36], and the remaining 2 studies (8%) targeted different acupuncture points [31,40] (Figure 1(b)).

In 15 studies, the Visual Analog Scale (VAS) was applied to evaluate pain levels in women with PD [17, 22–30, 35, 40–43] and further 3 studies used the numerical pain intensity rating (NRS) to assess pain intensity in PD [34, 39]. 2 studies used the McGill Pain Questionnaire (MPQ) and the pain rating index (PRI) as scales to assess pain [32, 33]. It is worth mentioning that 1 study used the present pain intensity (PPI) as an assessment indicator [33]. In addition, for the status of PD, 4 studies assessed patient symptoms on the Cox Menstrual Symptom Scale (CMSS) [22, 25, 35, 42] and 6 studies assessed patient psychological status using the Self-Rating Anxiety Scale (SAS) and Self-Rating Depression Scale (SDS) [17, 22, 25, 26, 36]. State-Trait Anxiety Inventory (SATI) and Beck Depression Inventory-II (BDI-II) have also been used to assess psychological conditions [32, 33]. 2 studies used the inclusion of hormonal indicators to assess the effect of acupuncture on patient hormone levels (Figure 1(f)) [32, 33].

17 studies (68%) all chose acupuncture Sanyinjiao point (SP6) as a therapeutic point [17, 22–28, 30, 32–34, 39, 41–43], 6 studies (24%) chose to explore suspension moxibustion of the Guanyuan point (RN4) [29, 31, 35, 37, 38], and 6 studies all compared changes in the brain before and after moxibustion. Furthermore, 2 studies (8%) explored the effect on brain function in patients with dysmenorrhea as a result of acupuncture on the Zusanli point (ST36) [36]. In this review, 22 studies (88%) chose to treat with a single acupoint, and the remaining 3 studies (12%) were treated with two acupoints together (Figure 1(a)) [41–43].

A total of 21 studies used magnetic resonance imaging (MRI) to investigate the brain response to acupuncture for migraines [17, 22, 24–33, 35–38, 40–42, 44]. Of these studies, 19 used fMRI [17, 22, 24–28, 30, 31, 33, 35–38, 40–43]. Moreover, 2 applied arterial spin labeling magnetic resonance imaging (ASL-MRI) [29, 32]. Four of the remaining studies employed positron emission tomography-computerized tomography (PET-CT) (Figure 1(e)) [23, 34, 39].

The standard analysis methods applied in fMRI were functional separation analysis (ALFF/fALFF; ReHo) (13 studies) [24–26, 30, 31, 35–38, 41–43] and functional integration analysis (FC, ICA) (7 studies) [17, 22, 27, 28, 33, 36, 40]. PET-CT by computational brain glucose metabolism analysis method (4 studies) was also applied [23, 34, 39]. One study used low-frequency fluctuation amplitude and local coherence analysis in functional segregation analysis [35]. In the present review, only one study chose programming analysis [29]. In addition, statistical parametric mapping (SPM) software, and DPARSF software were applied to evaluate the processed data (Figure 1(d)).

**Figure 2:**
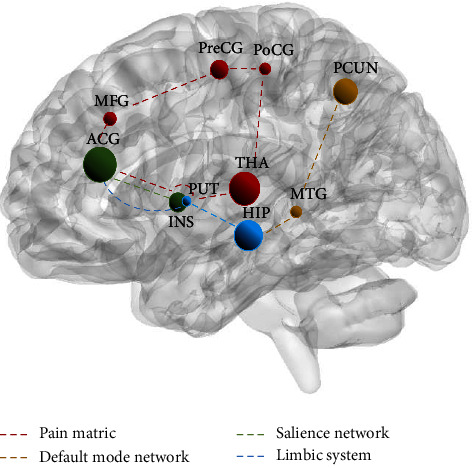
Main reported brain areas in patients with acupuncture-induced PD. The size of the nodes indicates the frequency of the brain areas. Each color dashed lines indicate the different modulation pathways of acupuncture. ACG: anterior cingulate gyrus; HIP: hippocampus; THA: thalamus; PCun: precuneus; INS: insula; PreCG: precentral gyrus; MFG: middle frontal gyrus; POCG: postcentral gyrus; MidTG: middle temporal gyrus; PUT: putamen.

Supplementary Table 4 shows the brain activation of acupuncture for PD.  Figure 2 depicts the most commonly encountered brain regions.

## 4. Discussion

This review summarizes the high-frequency brain areas of PD treated by acupuncture through the analysis of the previously mentioned studies and provides a reference for further research on the central mechanism and visual analysis of acupuncture for PD.

### 4.1. Brain Alterations of PD Patients

4 of the studies in this review referred to differences between the brains of dysmenorrheic patients and healthy individuals and found that dysmenorrheic patients exhibited altered brain function. Functional changes were usually manifested by decreased functional connectivity in the medial sensorimotor area, dorsolateral prefrontal, thalamus, insula, and postcentral gyrus, and increased functional connectivity in the medial orbitofrontal and hippocampus [22, 27]. The brain regions with abnormal alterations in ReHo values mainly involved the pain modulation network and the default network [36]. On brain glucose metabolism, brain areas associated with pain showed enhancement, such as the prefrontal, dorsolateral prefrontal, anterior cingulate gyrus, first somatosensory area, supplementary motor area, and first somatosensory area, and areas associated with emotion showed attenuation, such as temporal lobe, insula, caudate nucleus, hippocampal gyrus, corpus callosum, and hypothalamus [39]. These studies have laid the foundation for an in-depth study of the central mechanisms of acupuncture for PD.

### 4.2. Similarities and Differences in Brain Alterations in Patients with PD under the Effects of Acupuncture and Moxibustion

For PD, the number of acupuncture studies is much more than that of moxibustion. In terms of brain alterations in patients with PD, both acupuncture and moxibustion have been shown to activate analgesia-related brain regions to exert analgesic effects. In comparison to moxibustion, acupuncture studies also found that the activation of the nociceptive modulation system in the brain also activated the limbic system associated with emotion and pain perception [24, 25, 30, 41]. Moreover, research has reported that sham acupuncture activates nociceptive and affective brain areas in the retention state, suggesting the presence of a placebo effect of sham acupuncture [30]. This is not reported in moxibustion-related studies.

### 4.3. Brain Alterations in Patients with PD under Acupuncture Infection

In this review, 4 studies have demonstrated a decrease in glucose metabolism in the cerebral cortex and an increase in glucose metabolism in the limbic system in PD patients compared to healthy individuals [23, 34, 39]. Furthermore, from the perspective of analgesic mechanisms, verum and sham acupuncture modulate the perception of injurious input in PD patients by reducing cerebral blood flow through different brain response patterns [32]. In terms of functional connectivity, 4 reports indicated that the analgesic influence was achieved by activating the nociceptive modulation system in the brain during the postneedling effect. At the same time, the limbic system associated with pain perception was activated [24, 25, 30, 41]. This suggests that the central mechanism of acupuncture for PD may be mediated through the activation of the nociceptive modulation system and the cognitive-related limbic system.

### 4.4. Brain Network Alterations in Patients with PD under Acupuncture Infection

Acupuncture moderates a broadly located network of brain regions, and in this review, acupuncture of brain regions with high-frequency alterations in PD primarily included the anterior cingulate gyrus, hippocampus, thalamus, precuneus, insula, precentral gyrus, middle frontal gyrus, postcentral gyrus, middle temporal gyrus, putamen, and cerebellum as the main brain regions reported. They are mainly distributed in the pain matrix, default mode network (DMN), salience network, and limbic system. The anterior cingulate gyrus is not just an integral component of the “pain matrix” but also a critical area of the salience network and limbic system. The anterior cingulate cortex is engaged in analgesic regulation, pain memory, and emotional processing [45, 46]. The thalamus is an important relay station for transmitting injurious information to the cerebral cortex and an essential part of pain regulation [47, 48]. Notably, the precuneus plays an essential role in the DMN. Some researchers have found that reduced gray matter volume in the precuneus suggests increased thermal pain sensitivity [49]. However, this phenomenon is not present in every region of the pain matrix. The insula, as an essential node of the salience network as well as the pain matrix, improves individual brain-based pain sensitivity and increases thresholds for human pain, and is an important part of the brain involved in processing pain [50–52]. The cerebellum is commonly thought to be associated with movement, and the cerebellum supports experimental pain and chronic pain processing with its complex sensory, emotional, and neurocognitive aspects [53, 54].

#### 4.4.1. Pain Matrix Involved in the Brain Response to PD Acupuncture

The integrated study showed that regions of the pain matrix, which consist of the thalamus, precentral gyrus, anterior cingulate gyrus, insula, middle frontal gyrus, postcentral gyrus, and putamen, were involved in the brain response to acupuncture for PD (Figure 2 red dashed line). Activation of the pain matrix was related to the intensity of perceived pain within as well as between individuals [55]. Related studies suggested that matrix stimulation may be an effective method for reducing acute pain, therapeutic intervention with minimal side effects, and in the future, able to expand our pain management options for treating acute pain [56]. This review, through the modulatory effects of acupuncture on PD involving key parts of the pain matrix, suggested that acupuncture may work to treat PD by activating the pain matrix.

#### 4.4.2. Default Mode Network Involved in the Brain Response to Acupuncture in PD

The hippocampus, precuneus, and middle temporal gyrus are an important portion of the DMN system components involved in the brain response to acupuncture for PD (yellow dashed line in Figure 2). The DMN is associated with aspects of pain intensity, negative affect, or pain rumination. Related studies suggest that functional or structural abnormalities in relevant areas of the DMN may be related to central mechanisms of PD occurrence [57–59]. Therefore, acupuncture may have a therapeutic effect on PD by adjusting the distribution area of DMN.

#### 4.4.3. Salience Network Involved in Acupuncture Brain Response in PD

The anterior cingulate gyrus is the most mentioned brain region most mentioned in acupuncture for PD, and together with the insula, it is an important part of the salience network (green dashed line in Figure 2). Interestingly, studies showed that the salience network may be able to predict the onset of menstrual pain [17, 60]. It is predicted that the activity of the salience network could be monitored in the future to predict the onset of PD, and thus allow for precise acupuncture treatment of patients with PD.

#### 4.4.4. The Limbic System is Involved in the Brain Response to PD Acupuncture

In this review, the anterior cingulate gyrus, hippocampus, and putamen were high-frequency brain areas in the limbic system engaged in acupuncture for PD (blue dashed line in Figure 2). The limbic system is thought to be the system that processes motivational-emotional aspects, cognitive-evaluative aspects, and pain memory while mediating the analgesic effects of acupuncture [61, 62]. This suggests that the limbic system may regulate PD by modulating the function of corticolimbic brain regions involved in behavioral and emotional responses.

### 4.5. Limitations of the Study Design of Acupuncture Neuroimaging for PD

First, there is a dearth of studies exploring changes in the luteal phase of the brain concerning the timing of imaging acquisition. Can early intervention during the luteal phase, when peak estrogen promotes higher endometrial production of PGE2 relative to PGF2*α* in the premenstrual phase, alleviates dysmenorrhea if performed during the luteal phase? Brain dynamics are regulated by rhythmic variations in the concentration of female sex hormones throughout the menstrual cycle [63, 64]. The use of acupuncture stimulation during different phases of the menstrual cycle may bring about different changes in the brain that should be fully explored.

Second, the quality of life of PD patients should be focused on in the included studies by including relevant scales for evaluation. Pain is an unpleasant sensory and emotional experience [65]. Moreover, sex hormones were thought to be involved in modulating pain [66–68]. Therefore, PD should be viewed holistically, incorporating psychological, sex hormone levels, and quality of life scores.

Finally, most of the included studies used a single neuroimaging detection technique and a single analysis method. In the future, multiple neuroimaging techniques can be applied to detect dynamic brain activity in PD treated with acupuncture, and multimodal data analysis methods can be explored to gain fuller insight into the structural and functional changes in the brain brought about by PD treated with acupuncture.

## 5. Conclusion

The central mechanism of acupuncture for PD may be achieved by stimulation causing alterations in brain function and changes in different regional networks that activate the associated nociceptive and nociceptive cognitive systems. However, there is a need to continue to investigate the influence of acupuncture on brain alterations in PD patients during different phases of the menstrual cycle, and the influence of acupuncture on sex hormones and brain alterations in PD patients. Meanwhile, combining multiple imaging techniques and multiple data processing methods may become a novel direction to study the central mechanism of acupuncture for PD in the future.

## Data Availability

The raw data used to support the findings of this study are available from the corresponding author upon request.

## Supplementary Materials

Supplementary Materials Supplementary Materials. *Supplementary Figure 1.* The literature search and screening process. *Supplementary Figure 2.* Risk of bias assessment included in the study. *Supplementary Table 1.* Search strategy. *Supplementary Table 2.* The basic information of included studies. *Supplementary Table 3*. The study design. *Supplementary Table 4*. The neuroimaging information. *Supplementary Table 5.* The study details. *Figure 1.* The basic information of included studies. *Figure 2.* The most commonly encountered brain regions.
