# Supplementary material for: Revealing the Central Mechanism of Acupuncture for Primary Dysmenorrhea Based on Neuroimaging: A Narrative Review
Source: Pain Res Manag. 2023 Feb 18;2023:8307249. doi: 10.1155/2023/8307249 (PMC9966569; doi:10.1155/2023/8307249)
Supplement: Supplementary Materials — Supplementary Figure 1. The literature search and screening process. Supplementary Figure 2. Risk of bias assessment included in the study. Supplementary Table 1. Search strategy. Supplementary Table 2. The basic information of included studies. Supplementary Table 3. The study design. Supplementary Table 4. The neuroimaging information. Supplementary Table 5. The study details. Figure 1. The basic information of included studies. Figure 2. The most commonly encountered brain regions. [file 8307249.f1.zip › Revised_Supplementary_Table_1_search_strategy (1).docx]

Supplementary Material

# Supplementary Figures and Tables

## Supplementary Tables

**Table S1.** Full search strategy for each of the electronic databases queried

| **Databases** | **Coverage** | **Searches** | **Hits** |
| --- | --- | --- | --- |
| PubMed Database | Date of inception - Feb 16th, 2022 | 1. (((((((neuroimaging[MeSH Terms]) OR (neuroimaging[All fields])) OR ((neuroimaging[MeSH Terms]) OR (neuroimaging[All fields]))) OR (("magnetic resonance imaging"[MeSH Terms]) AND ("magnetic resonance imaging"))) OR ((Positron-Emission Tomography[MeSH Terms]) AND (Positron-Emission Tomography))) OR ((functional magnetic resonance imaging) OR (fMRI) OR (functional image))) OR ((Diffusion Tensor Imaging) OR (DTI))) OR ((structural magnetic resonance imaging) OR (sMRI))  2. (((((((((Dysmenorrheas[MeSH Terms]) OR (Pain, Menstrual)) OR (Menstrual Pain)) OR (Menstrual Pains)) OR (Pains, Menstrual)) OR (Menstruation, Painful)) OR (Menstruations, Painful)) OR (Painful Menstruation)) OR (Painful Menstruations)) OR (Primary dysmenorrhea)  3.((((((((((Acupuncture Therapy[MeSH Terms]) OR (acupuncture[MeSH Terms])) OR (Acupuncture Analgesia[MeSH Terms]))) OR (Acupuncture Therapy)) OR (Acupuncture)) OR (Acupuncture Points)) OR (acupressure)) OR (electroacupuncture)) OR (meridians)) OR (moxibustion) OR (deqi) OR (meridian) OR (acupoint)  4. #1 AND #2 AND #3 | 17 |
| EMBASE Database | Date of inception - Feb 16th, 2022 | #1. 'acupressure'/exp OR acupressure OR 'electroacupuncture'/exp OR electroacupuncture OR 'meridians'/exp OR meridians OR 'moxibustion'/exp OR moxibustion  #2. 'acupuncture therapy'/exp OR 'acupuncture therapy' OR 'acupuncture'/exp OR 'acupuncture' OR 'acupuncture points'/exp OR 'acupuncture points'  #3. acupoint$  #4. meridian$  #5. 'deqi'/exp OR deqi  #6. moxibustion$  #7. #1 OR #2 OR #3 OR #4 OR #5 OR #6  #8. 'dysmenorrhea'/exp OR 'dysmenorrhea' OR 'menstruations, painful':ti,ab,kw OR 'painful menstruations':ti,ab,kw OR 'pains, menstrual':ti,ab,kw OR 'dysmenorrheas':ti,ab,kw OR 'painful menstruation':ti,ab,kw OR 'pain, menstrual':ti,ab,kw OR 'menstrual pains':ti,ab,kw OR 'menstruation, painful':ti,ab,kw OR 'menstrual pain':ti,ab,kw OR 'primary dysmenorrhea':ti,ab,kw  #9. 'neuroimaging'/exp OR neuroimaging OR 'magnetic resonance imaging'/exp OR 'magnetic resonance imaging' OR 'mri'/exp OR mri OR 'structural magnetic resonance imaging'/exp OR 'structural magnetic resonance imaging' OR smri OR 'diffusion tensor imaging'/exp OR 'diffusion tensor imaging' OR 'dti' OR 'functional magnetic resonance imaging'/exp OR 'functional magnetic resonance imaging' OR 'fmri'/exp OR 'fmri' OR 'positron emission tomograph' OR 'pet'/exp OR pet  #10. #7 AND #8 AND #9 | 40 |
| Cochrane database | Date of inception - Feb 16th , 2022 | #1 “Acupuncture Therapy” or “Acupuncture” or “Acupuncture Points”  #2 acupressure or electroacupuncture or meridians or moxibustion  #3 acupoint$  #4 meridian$  #5 deqi  #6 moxibustion$  #7 #1 OR #2 OR #3 OR #4 OR #5 OR #6  #8 MeSH descriptor "Dysmenorrhea" explode all trees  #9 (Menstruations, Painful):ti,ab,kw OR (Painful Menstruations):ti,ab,kw OR (Pains, Menstrual):ti,ab,kw OR (Dysmenorrheas):ti,ab,kw OR (Painful Menstruation):ti,ab,kw OR (Pain, Menstrual):ti,ab,kw OR (Menstrual Pains):ti,ab,kw OR (Menstruation, Painful):ti,ab,kw OR (Menstrual Pain):ti,ab,kw OR (Primary dysmenorrhea):ti,ab,kw  #10 #8 OR #9  #11 neuroimaging OR ‘magnetic resonance imaging' OR mri OR 'structural magnetic resonance imaging' OR smri OR 'Diffusion Tensor Imaging' OR 'DTI' OR ‘functional magnetic resonance imaging’ OR ‘fmri’ OR 'positron emission tomograph' OR pet  #12 #7 AND #10 AND #11 | 10 |
| China National Knowledge Infrastructure (CNKI, Chinese Database) | Date of inception - Feb 16th, 2022 | (SU = 'dysmenorrhea' OR SU = 'primary dysmenorrhea') AND (SU = 'acupuncture' OR SU = 'electroacupuncture' OR SU = 'acupuncture manipulation' OR SU = 'de qi' OR SU = 'moxibustion' OR SU = 'acupuncture points' OR SU = 'meridians') AND (SU = 'neuroimaging' OR SU = 'brain imaging' OR SU = 'magnetic resonance technique' OR SU = ' functional MRI' OR SU = 'structural MRI' OR SU = 'diffusion tensor imaging' OR SU = ' positron emission tomograph ') | 15 |
| Wanfang Database  (WF, Chinese Database) | Date of inception - Feb 16th, 2022 | ((dysmenorrhea or primary dysmenorrhea) and (acupuncture or electroacupuncture or acupuncture manipulation or de qi or moxibustion or acupuncture points or meridians) and (neuroimaging or brain imaging or magnetic resonance techniques or functional MRI or structural MRI or diffusion tensor imaging or positron emission tomograph' )) | 35 |
| Chongqing VIP Database  (VIP, Chinese Database) | Date of inception - Feb 16th, 2022 | (R=( dysmenorrhea OR primary dysmenorrhea) OR M=(dysmenorrhea OR primary dysmenorrhea)) AND (R=(acupuncture OR electroacupuncture OR acupuncture OR de qi OR moxibustion OR acupuncture points OR meridians) OR M=(acupuncture OR electroacupuncture OR acupuncture OR de qi OR moxibustion OR acupuncture points OR meridians)) AND U=(neuroimaging OR brain imaging OR magnetic resonance techniques OR functional MRI OR structural MRI OR diffusion tensor imaging OR positron emission tomograph) | 6 |
| Chinese Biomedical Literature Database  (CBM, Chinese Database) | Date of inception - Feb 16th, 2022 | ("dysmenorrhea"[Common Field:Smart] OR "primary dysmenorrhea"[Common Field:Smart]) AND ("Acupuncture"[Common Field:Smart] OR "electroacupuncture"[Common Field:Smart] OR "acupuncture"[Common Field:Smart] OR "acupuncture"[Common Field:Smart] OR "de qi"[Common Field:Smart] OR "moxibustion"[Common Field:Smart] OR "acupuncture points"[Common Field:Smart] OR "meridians" [common field:smart]) AND ("neuroimaging" [all fields:smart] OR "brain imaging" [all fields:smart] OR "magnetic resonance technique" [all fields:smart] OR "functional MRI" [all fields:smart] OR "structural MRI" [all fields:smart] OR "diffusion tensor imaging" [all fields:smart] OR "positron emission tomograph" [all fields :smart]) | 7 |
