# Supplementary material for: Revealing the Central Mechanism of Acupuncture for Primary Dysmenorrhea Based on Neuroimaging: A Narrative Review
Source: Pain Res Manag. 2023 Feb 18;2023:8307249. doi: 10.1155/2023/8307249 (PMC9966569; doi:10.1155/2023/8307249)
Supplement: Supplementary Materials — Supplementary Figure 1. The literature search and screening process. Supplementary Figure 2. Risk of bias assessment included in the study. Supplementary Table 1. Search strategy. Supplementary Table 2. The basic information of included studies. Supplementary Table 3. The study design. Supplementary Table 4. The neuroimaging information. Supplementary Table 5. The study details. Figure 1. The basic information of included studies. Figure 2. The most commonly encountered brain regions. [file 8307249.f1.zip › Revised_Supplementary_Table_3_study_design_reference.docx]

**Supplementary Table. 3. The Study Design of the Included Studies.**

| Study  No. | Time of image capture | Intervention | |  |  | Controls | | Scale | |  |
| --- | --- | --- | --- | --- | --- | --- | --- | --- | --- | --- |
|  |  | **Manipulation** | **Sample size** | **Acupoint** | **Treatment** |  |  |  |  |  |
| S01([1](#_ENREF_1)) | Before and after moxibustion | SM | 18 | RN4 | Not mentioned | | Pre- vs Pos-Treatment | | VAS; CMSS | |
| S02([2](#_ENREF_2)) | Within 72 hours of the onset of menstruation | MA | 15 | SP6 | 3 menstrual cycles | | Active Acupoint vs. Inactive Acupoint | | VAS；CMSS；SAS；SDS | |
| S03([3](#_ENREF_3)) | Day 10 to 12 of the menstrual period | MA | 60 | ST36 | 1 menstrual cycles | | Patients vs. healthy people | | SF-MPQ;RSS;SAS;SDS | |
| S04([4](#_ENREF_4)) | Day 10 to 12 of the menstrual period | MA | 60 | ST36 | 1 menstrual cycles | | Patients vs. healthy people | | SF-MPQ;RSS;SAS;SDS | |
| S05([5](#_ENREF_5)) | Days 1-3 of the menstrual period | SM | 19 | RN4 | 2 menstrual cycles | | Moxibustion vs. Fake Moxibustion | | VAS | |
| S06([6](#_ENREF_6)) | At baseline and on the 5th day after the end of the menstrual period | MA | 6 | SP6 | 3 menstrual cycles | | Pre- vs Pos-Treatment | | VAS;COX | |
| S07([7](#_ENREF_7)) | Days 1-3 of the menstrual period | MA | 12 | SP6 | Not mentioned | | Active Acupoint vs. Inactive Acupoint | | VAS;COX | |
| S08([8](#_ENREF_8)) | Before and after moxibustion | SM | 20 | RN4 | Not mentioned | | Pre- vs Pos-Treatment | | Unspecified | |
| S09([9](#_ENREF_9)) | Before and after moxibustion | SM | 20 | RN4 | Not mentioned | | Pre- vs Pos-Treatment | | Unspecified | |
| S10([10](#_ENREF_10)) | Before and after moxibustion | SM | 20 | RN4/ RN8 | 3 menstrual cycles | | Different acupuncture points | | Unspecified | |
| S11([11](#_ENREF_11)) | Within 1-2 days of the onset of menstruation | MA | 6 | SP6 | 2 menstrual cycles | | VA vs SA | | NAS | |
| S12([12](#_ENREF_12)) | Within 1-2 days of the onset of menstruation | MA | 12 | SP6 | 2 menstrual cycles | | VA vs SA | | NAS | |
| S13([13](#_ENREF_13)) | Before and after moxibustion | SM | 60 | RN4 | Not mentioned | | Active Acupoint vs. Inactive Acupoint | | VAS | |
| S14([14](#_ENREF_14)) | Day 1-2 of menstruation | MA | 16 | SP6/ SP8 | Not mentioned | | Pre- vs Pos-Treatment | | VAS | |
| S15([15](#_ENREF_15)) | Day 1-2 of menstruation | MA | 16 | SP6/ SP8 | Not mentioned | | Pre- vs Pos-Treatment | | VAS | |
| S16([16](#_ENREF_16)) | Day 1-2 of menstruation | MA | 16 | SP6 | Not mentioned | | VA vs SA | | VAS | |
| S17([17](#_ENREF_17)) | Day 1-2 of menstruation | MA | 6 | SP6 | 2 menstrual cycles | | VA vs SA | | NAS | |
| S18([18](#_ENREF_18)) | Day 1-2 of menstruation | MA | 6 | SP6/ SP8 | 1 menstrual cycles | | VA vs SA | | VAS;CMSS | |
| S19([19](#_ENREF_19)) | Day 1-2 of menstruation | MA | 12 | SP6 | 3 menstrual cycles | | Active Acupoint vs. Inactive Acupoint | | VAS;CMSS;SAS;SDS | |
| S20([20](#_ENREF_20)) | Day 1-3 of menstruation | MA | 16 | SP6 | 1 menstrual cycles | | Active Acupoint vs. Inactive Acupoint | | VAS;CMSS;SAS;SDS | |
| S21([21](#_ENREF_21)) | Day 1-3 of menstruation | MA | 20 | SP6 | 1 menstrual cycles | | Patients vs. healthy people | | VAS | |
| S22([22](#_ENREF_22)) | Day 5 to 12 of the menstrual period | MA | 23 | SP6 | 8 weeks | | VA vs SA | | MPQ;PRI;STAI;BDI II; Estrogen; Progesterone; Testosterone | |
| S23([23](#_ENREF_23)) | Day 5 to 12 of the menstrual period | MA | 34 | SP6 | 8 weeks | | VA vs SA | | MPQ;PRI;PPI;STAI;BDI II; Estrogen; Progesterone; Testosterone | |
| S24([24](#_ENREF_24)) | Day 1-3 of menstruation | MA | 34 | SP6 | 8 weeks | | VA vs SA | | VAS | |
| S25([25](#_ENREF_28)) | At baseline and on days 12-16 of the menstrual period | MA | 35 | SP6 | 3 menstrual cycles | | VA vs SA | | VAS;SAS;SDS | |

**Abbr.**

VA. Verum Acupuncture; SA. Sham Acupuncture;

MA. Manual Acupuncture; SA. Suspension Moxibustion

VAS. Visual Analogue Scale; NRS. Numerical Rating Scale;

CMSS. the Cox Menstrual Symptom Scale; SAS. Self-rating Anxiety Scale;

SDS. Self-rating Depression Scale; MPQ. McGill Pain Questionnaire;

PRI. Pain Rating Index; PPI. Present Pain Intensity;

STAI. State-trait Anxiety Inventory; BDI-II. Beck Depression Inventory-II.

**Reference.**

1. Zhou X.C. Based on rs-fMRI technology to study the brain function regulation mechanism of the heat-sensitive state of Guanyuan point in patients with primary dysmenorrhea with moxibustion. (Jiangxi University of Traditional Chinese Medicine, 2021).

2. Yu S. Y. Frequency-specific alteration of functional connectivity density in primary dysmenorrhea modulation effect of acupuncture. (Chengdu University of Traditional Chinese Medicine, 2019).

3. Jin L.M. The central mechanism of the immediate effect of acupuncture based on functional magnetic resonance imaging. (Xi'an University of Electronic Science and Technology, 2017).

4. Jin L.M. The central mechanism of the immediate effect of acupuncture based on functional magnetic resonance imaging. (Xi'an University of Electronic Science and Technology, 2017).

5. Chen Z.Y. Study on the central analgesic mechanism of moxibustion for primary dysmenorrhea based on arterial spin-labeled functional magnetic resonance imaging. (Beijing University of Traditional Chinese Medicine, 2017).

6. Chen X.Z. Central mechanism of acupuncture in patients with primary dysmenorrhea based on PET-CT. (Chengdu University of Traditional Chinese Medicine, 2015).

7. Luo X. Central mechanism of instant analgesia effect of acupuncture to primary dysmenorrhea patients based on fMRI. (Chengdu University of Traditional Chinese Medicine, 2015).

8. Song Y.E. et al. Moxibustion of Guan Yuan point for primary dysmenorrhea :a resting-state functional magnetic resonance imaging study. Journal of Chongqing Medical University 37, 753-758 (2012).

9. Song Y.E. et al. Regional homogeneity of primary dysmenorrheal :a resting-state fMRI study. World Scientific Research and Development 34, 501-505 (2012).

10. Song Y.E. Clinical and central analgesic mechanism research of therapeutic effect of thermal moxibustion therapies of Guan Yuan point on menstruation associated symptoms. (Chongqing Medical University, 2012).

11. Gong P. Study of acupuncture action on local and central modulation mechanisms. (Huazhong University of Science and Technology, 2006).

12. Gong P. Study of acupuncture action on local and central modulation mechanisms. (Huazhong University of Science and Technology, 2006). 13.

13. Xie D.Y.et al. Study on effects of moxibustion at heat-sensitized Guanyuan(RN 4) on brain functional connective network in patients with primary dysmenorrhea. World TCM 14, 1922-1928+1935 (2019).

14. Su C.G. et al. Study on the functional regions of acupuncture treatment for primary dysmenorrhea in brain by the technology of resting-state functional magnetic resonance imaging. Sichuan Traditional Chinese Medicine 34, 165-168 (2016).

15. Su C.G. To study the functional regions of acupuncture treatment on primary dysmenorrhea in brain by the technology of resting-state functional brain magnetic resonance imaging. (The Second Hospital of Henan Provincial University of Traditional Chinese Medicine, 2012).

16. Li.H. To study the functional regions of acupuncture at Sanyinjiao(SP6) treatment on primary dysmenorrhea. (Henan College of Traditional Chinese Medicine, 2014).

17. Gong P et al. Effect of acupuncture at Sanyinjiao(SP6) on glucose metabolism in the patients of dysmenorrhea. Chinese Acupuncture 51-55 (2006). 18.

18. Chen X.Y. Analgesic effect evaluation and brain function mechanism discovery of acupuncture treatment on primary dysmenorrhea. ( The Second Hospital of Henan University of Traditional Chinese Medicine, 2013.)

19. Wang.Y.-X. A rs-fMRI study on the central mechanism through acupuncture treatment in primary dysmenrrhea. (Chengdu University of Traditional Chinese Medicine, 2016).

20. Zhang Q. A study of the influence on puncturing sanyinjiao for primary dysmenorrhea in resting-state brain function. (Chengdu University of Traditional Chinese Medicine, 2017).

21. Zhang Q. Using hippocampus as the seed to study the change of rs-fc in primary dysmenorrhea patients by needing SP6. (Chengdu University of Traditional Chinese Medicine, 2017).

22. Peng, S.-L. et al. Analgesia Effect of Verum and Sham Acupuncture Treatments in Primary Dysmenorrhea: A MRI Pilot Study. J Pers Med 11, 1244 (2021).

23. Tu, C.-H. et al. Acupuncture Treatment Associated with Functional Connectivity Changes in Primary Dysmenorrhea: A Resting State fMRI Study. J Clin Med 10, 4731 (2021). 24.

24. Wang, Y. et al. Immediate Analgesic Effect of Acupuncture in Patients With Primary Dysmenorrhea: A fMRI Study. Front Neurosci 15, 647667 (2021).

25. Yu, S. et al. Resting-State Functional Connectivity Patterns Predict Acupuncture Treatment Response in Primary Dysmenorrhea. Front Neurosci 14, 559191 (2020).
