# Supplementary material for: Revealing the Central Mechanism of Acupuncture for Primary Dysmenorrhea Based on Neuroimaging: A Narrative Review
Source: Pain Res Manag. 2023 Feb 18;2023:8307249. doi: 10.1155/2023/8307249 (PMC9966569; doi:10.1155/2023/8307249)
Supplement: Supplementary Materials — Supplementary Figure 1. The literature search and screening process. Supplementary Figure 2. Risk of bias assessment included in the study. Supplementary Table 1. Search strategy. Supplementary Table 2. The basic information of included studies. Supplementary Table 3. The study design. Supplementary Table 4. The neuroimaging information. Supplementary Table 5. The study details. Figure 1. The basic information of included studies. Figure 2. The most commonly encountered brain regions. [file 8307249.f1.zip › Revised_Supplementary_Table_5_Study_Details.docx]

**Supplementary Table. 5. The Study Details of the Included Studies.**

| Study  No. | Intervention | |  |  | Scanning Results | |
| --- | --- | --- | --- | --- | --- | --- |
|  | **Manipulation** | **Sample size** | **Acupoint** | **Treatment** |  |  |
| S01([1](#_ENREF_1)) | SM | 18 | RN4 | Not mentioned | | After Moxibustion Treatment:  ↑ALFF: MFG, PreCun;  ↓ALFF: Cuneate lobe, Supraoccipital Gyrus, Middle Occipital Gyrus  ↑ReHo: MFG;STG  ↓ReHo: PreCG,POCG |
| S02([2](#_ENREF_2)) | MA | 15 | SP6 | 3 menstrual cycles | | After Acupuncture Treatment:  ↑FC: DMN, Medial Control Network, Hippocampal Network  ↓FC: Primary Sensorimotor Network, Lateral Control Network |
| S03([3](#_ENREF_3)) | MA | 60 | ST36 | 1 menstrual cycles | | Menstrual period:  Abnormal brain areas: Pain Management Network; DMN  Non-menstrual period:  Abnormal brain areas: Sensory Motion zones and DMN |
| S04([4](#_ENREF_4)) | MA | 60 | ST36 | 1 menstrual cycles | | Abnormal brain areas: DMN, Visual Network, Highlight Network, Executive Control Network |
| S05([5](#_ENREF_5)) | SM | 19 | RN4 | 2 menstrual cycles | | After Moxibustion Treatment:  ↑CBF: Left Angular Gyrus, Left Anterior Cingulate Gyrus and Paracingulate Gyrus, Left Posterior Cingulate Gyrus, Left Middle Frontal Gyrus Medial to Superior Frontal Gyrus, Left Inferior Parietal Marginal Angular Gyrus, Left Supramarginal Gyrus |
| S06([6](#_ENREF_6)) | MA | 6 | SP6 | 3 menstrual cycles | | After Acupuncture Treatment:  ↑Brain Glucose Metabolism: STG, SMG, MidTG, PreCun, Central Temporal Zone |
| S07([7](#_ENREF_7)) | MA | 12 | SP6 | Not mentioned | | After Acupuncture Treatment:  Meridian points Group: THA, Prefrontal,Orbitofrontal cortex,ACG, HIP,INS, Temporal lobe  Non-menstrual period: PreCun, Middle Orbital Frontal Gyrus; Caudate Nucleus |
| S08([8](#_ENREF_8)) | SM | 20 | RN4 | Not mentioned | | After Moxibustion Treatment:  ↑ALFF: CG,INS, PCun, POCG, Sulcus Cerebri , STG , MidTG，PreCG，MFG，IFG  ↓ALFF: Occipital Lobe , SFG , MFG, STG, Posterior Cerebellar Slopes |
| S09([9](#_ENREF_9)) | SM | 20 | RN4 | Not mentioned | | After Moxibustion Treatment:  ↑ReHo: STG,MTG,MFG, PreCG,SMG,ACC  ↓ReHo: IFG, THA, Occipital Lobe |
| S10([10](#_ENREF_10)) | SM | 20 | RN4/ RN8 | 3 menstrual cycles | | After Moxibustion Treatment:  ↑ReHo: STG, MTG,ACG,CG,MTG, Medial Frontal Gyrus, PreCG,POCG  ↓ReHo: Cuneus, Midbrain, THA, Occipital Lobe |
| S11([11](#_ENREF_11)) | MA | 6 | SP6 | 2 menstrual cycles | | After Acupuncture Treatment:  ↑Brain Glucose Metabolism: Leguminous nucleus (pallidum, shell), cerebellum, INS, THA, paracentral lobule, AMYG , Midbrain Substantia Nigra, SII, Parahippocampal gyrus, ACC, Hypothalamus, Papillary Bodies  ↓Brain Glucose Metabolism: Cerebral cortex |
| S12([12](#_ENREF_12)) | MA | 12 | SP6 | 2 menstrual cycles | | After Acupuncture Treatment:  Patients:  ↑Brain Glucose Metabolism: SⅡ, Limbic system, Cerebellum  Healthy People:  ↑Brain Glucose Metabolism: Cerebral Cortex |
| S13([13](#_ENREF_13)) | SM | 60 | RN4 | Not mentioned | | Thermal Moxibustion Group：  ↑FC: Brainstem-Cerebellum  ↓FC: Brain - white matter areas of the brain - insula - frontal lobe |
| S14([14](#_ENREF_14)) | MA | 16 | SP6/ SP8 | Not mentioned | | After Acupuncture Treatment:  ↓ALFF:CG,ACG, HIP,THA,INS, Prefrontal Cortex, Lenticular nucleus, Midbrain, Pons |
| S15([15](#_ENREF_15)) | MA | 16 | SP6/ SP8 | Not mentioned | | After Acupuncture Treatment:  ↑ALFF:MFG, SFG, ACG ,CC  ↓ALFF: CG, ACG,INS, HIP, THA, Nucleus Accumbens, Midbrain,Pontine Brain, Cerebellum |
| S16([16](#_ENREF_16)) | MA | 16 | SP6 | Not mentioned | | VA Group:  ↑ALFF: HIP,STG, ACG, Caudate Nucleus, SFG, MFG, Corpus callosum  ↓ALFF: Pons, HIP, Caudate Nucleus, INS, THA, ACG, SFG |
| S17([17](#_ENREF_17)) | MA | 6 | SP6 | 2 menstrual cycles | | After Acupuncture Treatment:  ↑Brain Glucose Metabolism: Leguminous nucleus (pallidum, shell), Cerebellum,Insula, Dorsal Thalamus, Paracentral Lobule,Amygdala, Midbrain Substantia Nigra, SII, Hippocampal gyrus, Anterior Cingulate Gyrus, Hypothalamus Papillae  ↓Brain Glucose Metabolism: Cerebral cortex |
| S18([18](#_ENREF_18)) | MA | 6 | SP6/ SP8 | 1 menstrual cycles | | VA Group:  ↑ALFF: Occipital Lobe, POCG, Supramarginal Lobule, SMG, AG, PreCun, CG |
| S19([19](#_ENREF_19)) | MA | 12 | SP6 | 3 menstrual cycles | | Active Acupoint:  ↑ALFF: Cuneate Leaves, POCG, Parietal Leaflets  ↓ALFF: MFG, ACG, Cerebellar Earthworm, SFG, MFG, Paracentral Lobule |
| S20([20](#_ENREF_20)) | MA | 16 | SP6 | 1 menstrual cycles | | Active Acupoint Group vs. Inactive Acupoint Group  ↑ReHo: Lateral Prefrontal Cortex, ACC,DPFC  ↓ReHo: THA, Lingual Gyrus |
| S21([21](#_ENREF_21)) | MA | 20 | SP6 | 1 menstrual cycles | | Patients vs. healthy people:  ↑FC: Seahorse-Orbital Gyrus  ↓FC: THA, INS, POCG,HIP, Motor Cortex  Pre- vs Pos-Treatment:  ↑FC: HIP,THA, PreCG, POCG, AG  ↓FC: Orbital Frontal Gyrus |
| S22([22](#_ENREF_22)) | MA | 23 | SP6 | 8 weeks | | SA Group:  ↓CBF: Ventral Medial Prefrontal Cortex, Caudate Nucleus,INS  VA Group:  ↓CBF:ACC |
| S23([23](#_ENREF_23)) | MA | 34 | SP6 | 8 weeks | | VA Group vs SA Group:  ↓FC: periaqueductal gray matter and the regions associated with affective pain modulation and attention-related pain modulation |
| S24([24](#_ENREF_24)) | MA | 34 | SP6 | 8 weeks | | VA Group:  ↑FC: ACC, supplementary motor areas |
| S25([25](#_ENREF_28)) | MA | 35 | SP6 | 3 menstrual cycles | | ↑FC(VA Group>SA Group): DPMS-SMN  ↓FC: (VA Group <SA Group): SMN-SN,DMN-SN |

**Abbr.**

VA. Verum Acupuncture; SA. Sham Acupuncture;

MA. Manual Acupuncture; SM. Suspension Moxibustion

ALFF. Amplitude of Low Frequency Fluctuations; FC. Functional Connectivity;

ReHo. Regional Homogeneity; ICA. Independent Components Analysis;

MFG. Middle frontal gyrus ; STG. Superior temporal gyrus;

PreCun. Precuneus; PAG. Periaqueductal Gray;

PreCG. Pre-central gyrus; POCG. Post-central Gyrus;

SMG. Suprafrontal Fyrus; MidTG. Middle Temporal Gyrus;

THA. Thalamus; HIP. Hippocampus;

INS. Insula; ACC. Anterior Cingulate Cortex;

CG. Cingulate Gyrus; IFG. Inferior Frontal Gyrus;

MTG. Middle Temporal Gyrus; ACG. Anterior cingulate gyrus;

AMYG. Amygdala; SII. Somatosensory Region II;

SFG. Suprafrontal Gyrus; AG. Angular Gyrus;

dPFC. Dorsolateral Prefrontal Cortex; DPMS. Descending Modulation Pathways

DMN. Default Mode Network; FPN. Frontoparietal Network

SMN. Sensorimotor Network; SN. Salience Network

**Reference.**

1. Zhou X.C. Based on rs-fMRI technology to study the brain function regulation mechanism of the heat-sensitive state of Guanyuan point in patients with primary dysmenorrhea with moxibustion. (Jiangxi University of Traditional Chinese Medicine, 2021).

2. Yu S. Y. Frequency-specific alteration of functional connectivity density in primary dysmenorrhea modulation effect of acupuncture. (Chengdu University of Traditional Chinese Medicine, 2019).

3. Jin L.M. The central mechanism of the immediate effect of acupuncture based on functional magnetic resonance imaging. (Xi'an University of Electronic Science and Technology, 2017).

4. Jin L.M. The central mechanism of the immediate effect of acupuncture based on functional magnetic resonance imaging. (Xi'an University of Electronic Science and Technology, 2017).

5. Chen Z.Y. Study on the central analgesic mechanism of moxibustion for primary dysmenorrhea based on arterial spin-labeled functional magnetic resonance imaging. (Beijing University of Traditional Chinese Medicine, 2017).

6. Chen X.Z. Central mechanism of acupuncture in patients with primary dysmenorrhea based on PET-CT. (Chengdu University of Traditional Chinese Medicine, 2015).

7. Luo X. Central mechanism of instant analgesia effect of acupuncture to primary dysmenorrhea patients based on fMRI. (Chengdu University of Traditional Chinese Medicine, 2015).

8. Song Y.E. et al. Moxibustion of Guan Yuan point for primary dysmenorrhea :a resting-state functional magnetic resonance imaging study. Journal of Chongqing Medical University 37, 753-758 (2012).

9. Song Y.E. et al. Regional homogeneity of primary dysmenorrheal :a resting-state fMRI study. World Scientific Research and Development 34, 501-505 (2012).

10. Song Y.E. Clinical and central analgesic mechanism research of therapeutic effect of thermal moxibustion therapies of Guan Yuan point on menstruation associated symptoms. (Chongqing Medical University, 2012).

11. Gong P. Study of acupuncture action on local and central modulation mechanisms. (Huazhong University of Science and Technology, 2006).

12. Gong P. Study of acupuncture action on local and central modulation mechanisms. (Huazhong University of Science and Technology, 2006). 13.

13. Xie D.Y.et al. Study on effects of moxibustion at heat-sensitized Guanyuan(RN 4) on brain functional connective network in patients with primary dysmenorrhea. World TCM 14, 1922-1928+1935 (2019).

14. Su C.G. et al. Study on the functional regions of acupuncture treatment for primary dysmenorrhea in brain by the technology of resting-state functional magnetic resonance imaging. Sichuan Traditional Chinese Medicine 34, 165-168 (2016).

15. Su C.G. To study the functional regions of acupuncture treatment on primary dysmenorrhea in brain by the technology of resting-state functional brain magnetic resonance imaging. (The Second Hospital of Henan Provincial University of Traditional Chinese Medicine, 2012).

16. Li.H. To study the functional regions of acupuncture at Sanyinjiao(SP6) treatment on primary dysmenorrhea. (Henan College of Traditional Chinese Medicine, 2014).

17. Gong P et al. Effect of acupuncture at Sanyinjiao(SP6) on glucose metabolism in the patients of dysmenorrhea. Chinese Acupuncture 51-55 (2006). 18.

18. Chen X.Y. Analgesic effect evaluation and brain function mechanism discovery of acupuncture treatment on primary dysmenorrhea. ( The Second Hospital of Henan University of Traditional Chinese Medicine, 2013.)

19. Wang.Y.-X. A rs-fMRI study on the central mechanism through acupuncture treatment in primary dysmenrrhea. (Chengdu University of Traditional Chinese Medicine, 2016).

20. Zhang Q. A study of the influence on puncturing sanyinjiao for primary dysmenorrhea in resting-state brain function. (Chengdu University of Traditional Chinese Medicine, 2017).

21. Zhang Q. Using hippocampus as the seed to study the change of rs-fc in primary dysmenorrhea patients by needing SP6. (Chengdu University of Traditional Chinese Medicine, 2017).

22. Peng, S.-L. et al. Analgesia Effect of Verum and Sham Acupuncture Treatments in Primary Dysmenorrhea: A MRI Pilot Study. J Pers Med 11, 1244 (2021).

23. Tu, C.-H. et al. Acupuncture Treatment Associated with Functional Connectivity Changes in Primary Dysmenorrhea: A Resting State fMRI Study. J Clin Med 10, 4731 (2021). 24.

24. Wang, Y. et al. Immediate Analgesic Effect of Acupuncture in Patients With Primary Dysmenorrhea: A fMRI Study. Front Neurosci 15, 647667 (2021).

25. Yu, S. et al. Resting-State Functional Connectivity Patterns Predict Acupuncture Treatment Response in Primary Dysmenorrhea. Front Neurosci 14, 559191 (2020).
